# Ligand-Controlled Phonon Dynamics in CsPbBr_3_ Nanocrystals Revealed by Machine-Learned Interatomic Potentials

**DOI:** 10.1021/jacsau.6c00434

**Published:** 2026-04-27

**Authors:** Seungjun Cha, Chen Wang, Victor Fung, Guoxiang Hu

**Affiliations:** † School of Materials Science and Engineering, 1372 Georgia Institute of Technology, Atlanta, Georgia 30332, United States; ‡ Department of Chemistry and Biochemistry, Queens College, City University of New York, New York, New York 11367, United States; § The Graduate Center, 14772 City University of New York, New York, New York 10016, United States; ∥ School of Computational Science and Engineering, 1372 Georgia Institute of Technology, Atlanta, Georgia 30332, United States; ⊥ School of Chemistry and Biochemistry, 1372 Georgia Institute of Technology, Atlanta, Georgia 30332, United States

**Keywords:** halide perovskites, nanocrystals, phonons, surface ligands, machine learning potentials, molecular dynamics

## Abstract

Halide perovskite nanocrystals are leading candidates for next-generation optoelectronics, yet the role of surface ligands in controlling their phonon dynamics remains poorly understood. These lattice dynamics critically govern energy up-conversion, phonon-assisted anti-Stokes emission, and nonradiative relaxation. Conventional ab initio methods, while accurate, are computationally infeasible for experimentally relevant nanocrystal sizes that require thousands of atoms to capture realistic ligand shells and dynamic disorder. Here, we introduce a machine-learned interatomic potential fine-tuned on small CsPbBr_3_ nanocrystals with diverse ligands, enabling accurate prediction of ligand-induced phonon properties far beyond the spatial and temporal scales of ab initio methods. We find that both cationic and anionic ligands systematically redshift Pb–Br–Pb stretching modes while blueshifting the PbBr_6_^4–^ octahedral rotation mode, with stronger overall effects for anionic passivation. Notably, anionic ligands stiffen the rotation mode nonmonotonically with respect to the ligand binding energy. The nanocrystal models also reveal a strong site dependence of these phonon-mode shifts, with corner and edge sites showing the largest response. Our findings reveal important roles of cationic and anionic ligands in modulating key dynamic modes of halide perovskite nanocrystals associated with detrimental nonradiative losses, offering mechanistic insights and design principles for high-performance perovskite nanocrystal optoelectronics.

## Graphical abstract

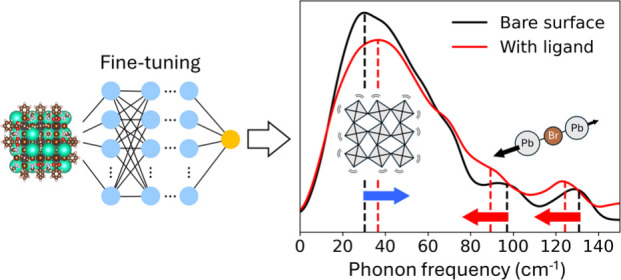

## Introduction

Halide perovskite nanocrystals (NCs) have emerged as the next-generation optoelectronic materials due to narrow emission line width, high defect tolerance, and low-cost solution-processability, which enables precise control over compositions and sizes for property tuning.^1,2^ In particular, colloidal NCs with sizes in the quantum confinement regime largely benefit from enhanced exciton binding energies and carrier localization,^3^ making them attractive candidates for light-emitting applications such as LEDs,^4^ lasers,^5^ and single-photon sources.^6^ Colloidal synthesis of halide perovskite NCs typically employs organic ligands, which cap the surface of the inorganic core to provide colloidal stability in solvents. These ligands play a pivotal role in determining the optoelectronic performance of the NCs. Surface ligands are essential for passivating trap states in the bandgap (i.e., deep traps), which promote nonradiative recombination pathways that harm photoluminescence quantum yield (PLQY). Deep traps predominantly arise from surface defects^7,8^ and become especially detrimental to NCs with a high surface-to-volume ratio,^9^ hindering near-unity PLQY ideal for light-emitters.

More recently, the role of surface ligands in controlling phonon dynamics has been increasingly recognized. For example, conjugated molecular multipods have been shown to suppress dynamic disorder in FAPbBr_3_ by stiffening the lattice through hydrogen bonding and van der Waals interactions with the surface FA molecules, observed as a blueshift of the low-energy PbBr_6_^4–^ rotation mode.^10^ Low-energy phonon modes in CsPbBr_3_ such as rotation, bending and stretching modes of Pb and Br, which directly couple to surface ligands, have been identified to govern processes such as energy up-conversion^11^ and phonon-assisted anti-Stokes emission.^12^ At the same time, lattice phonon modes are directly responsible for electron–phonon coupling, which results in nonradiative relaxation pathways.^13,14^ These processes have direct implications for radiative efficiency, performance, and applications of perovskite nanocrystal optoelectronics. Thus, fundamental understanding of nanocrystal surfaces, particularly how ligands affect low-energy phonons, emerges as a key challenge for designing these unique materials systems. Yet, despite their importance, the theoretical understanding of ligand-controlled phonon dynamics remains limited, in part due to the lack of computationally tractable methods.

Conventional ab initio studies model halide perovskite NCs as periodic slabs,^7,15–17^ in which a large vacuum is added along one axis while maintaining periodicity along the others. While computationally efficient, slab models are inherently unsuitable for capturing the complexity of NCs, which are quantum-confined in all three dimensions. As a result, they fail to capture key properties including size effect, site-dependent properties (at corners and edges), and interactions between opposing surfaces present in a finite system. By contrast, realistic NC models naturally account for these effects but come with a prohibitive computational cost: due to the lack of periodicity, the number of atoms scales exponentially with NC size, making ab initio methods such as density functional theory (DFT) impractical beyond modest spatial and temporal scales. The incorporation of surface ligands exacerbates the problem. For example, a realistic 4 nm CsPbBr_3_ NC model capped with 50% surface coverage of methylammonium and benzoate has ∼5000 atoms, which is far beyond the tractable limit for conventional DFT. The compute-heavy nature of phonon calculations and molecular dynamics forces this limit to be even lower. Consequently, phonon studies using NC models have largely been restricted to ligand-free systems^13^ or those with limited coverage.^18^ This calls for a more computationally efficient alternative to fully capture the effect of surface ligands in realistic NCs.

To overcome these limitations, we turn to universal pretrained machine-learned interatomic potentials (MLIPs) to achieve near-DFT accuracy at a fraction of computational cost. Many existing universal MLIPs^19–23^ are largely pretrained on bulk crystals, readily available as standardized data sets such as OMat24,^24^ MPtrj,^22^ and Alex-MP-20.^25^ Bulk phase data sets are easier to generate at scale under periodic boundary conditions, with well-defined unit cells, compositions, and symmetries. In contrast, finite nanocrystal systems introduce substantial configurational complexity, asymmetry, and degrees of freedom arising from the interfaces, hindering scalability and public availability. In this work, we generated a training data set comprised of small CsPbBr_3_ NC models with DFT-feasible sizes and diverse ligands. We report that universal pretrained MLIPs can be effectively fine-tuned on this data set to extrapolate well outside the length scale represented in the training regime. We then systematically compute the phonon density of states of the representative NC models across different sizes to reveal ligand-controlled phonon dynamics in CsPbBr_3_ NCs. We discuss the mode and site-selective effect of cationic and anionic ligands on characteristic Pb–Br dynamics. We highlight the nonmonotonic relationship between anionic ligand binding energy and the stiffening of the PbBr_6_^4–^ octahedral rotation mode, which is further studied using MLIP-based molecular dynamics simulations. These results provide insights into the design principles for tailoring surface chemistry to control lattice disorder in halide perovskite optoelectronics.

## Results and Discussion

### Fine-Tuning a Universal Pretrained Machine-Learned Interatomic Potential

We first constructed a systematic set of CsPbBr_3_ NC models with varying supercell sizes, surface passivation types, and ligand coverage (Figure 1). The three representative cases were (1) bare NCs, (2) methylammonium (MA)-capped NCs, and (3) more realistic mixed-ligand NCs capped with both MA and anionic ligands. (Table S1 provides a full list of ligands represented in the training data set). All NC models were constructed with Cs–Br surface termination, as several studies strongly supported Cs-exposed surface, where a fraction of Cs^+^ sites are replaced by the ammonium cations introduced during typical colloidal synthesis.^16,26,27^ Here, we use MA to represent widely used ammonium-derivatives such as oleylammonium. Under Cs–Br termination, net positive charge arises from the excess Cs atoms on the surface. Therefore, to enforce strict charge neutrality, we first calculate the net positive charge of each NC by summing over the formal ionic charges of Cs^+^, Pb^2+^, and Br^–^. Due to significant site-dependent binding energies of Cs (Table S2), we first remove Cs atoms from the corner sites, which have the smallest binding energy, and then randomly remove from the remaining surface sites as needed to maintain charge neutrality. In ligand-capped models, MA and anionic ligands randomly replaced surface Cs and Br atoms, respectively, until reaching a specified ligand coverage. Here, ligand coverage was separately defined for each ligand as the fraction of respective surface sites substituted; for consistency, the same nominal coverage was applied to both species throughout this work. To capture an even wider phase space and local environments during training, atomic positions were randomly perturbed in proportion to their covalent radii. The train and test data sets were then generated from the geometry relaxation trajectories of the NC models using Vienna Ab initio Simulation Package (VASP),^28^ naturally yielding many near-equilibrium structures favorable for finite-difference phonon calculations. More details on the model construction and train set generation can be found in Supporting Information.

**Figure 1:**
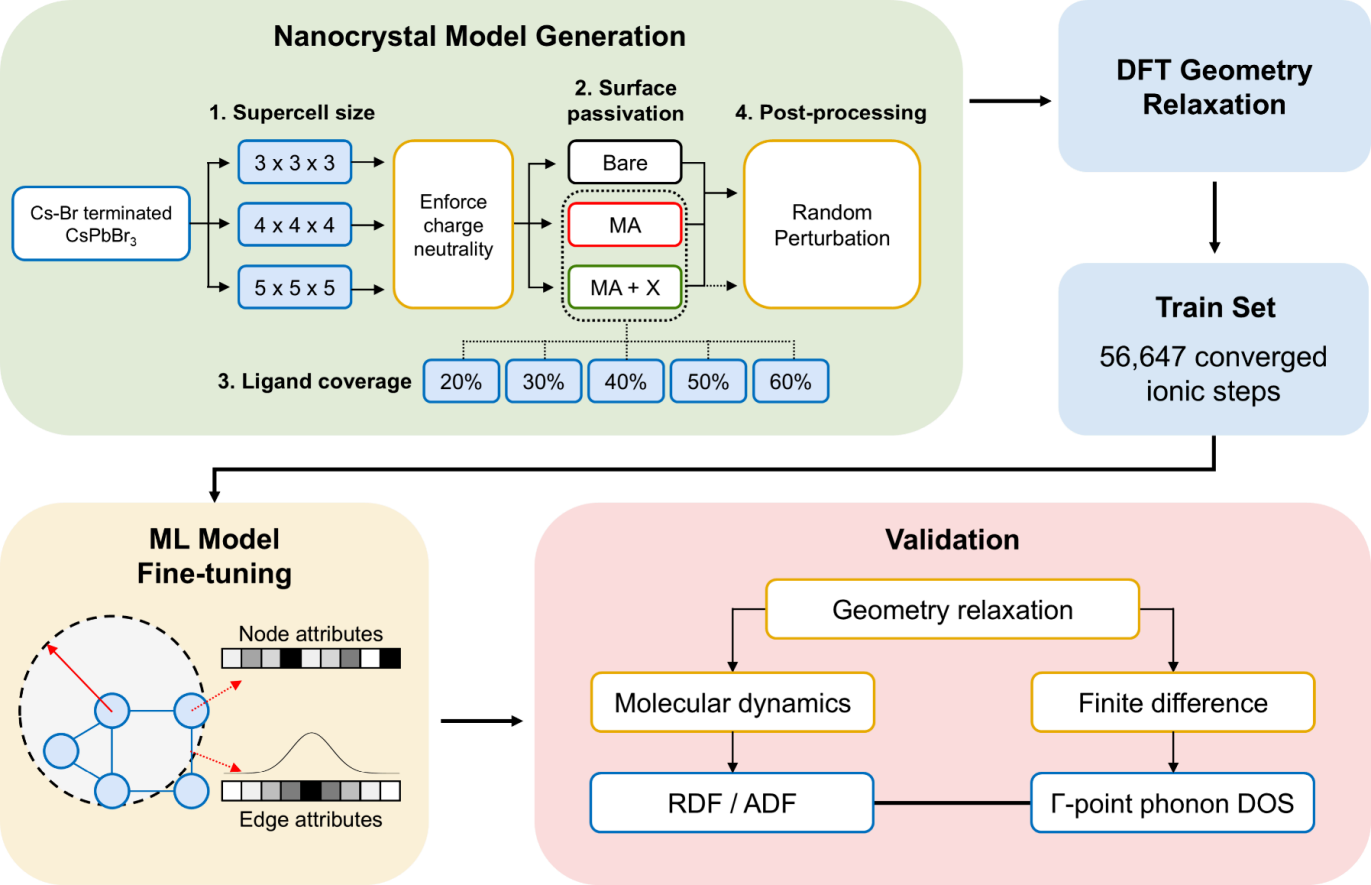
Overview of this work. NC models were generated by varying (1) supercell size, (2) surface passivation scheme, and (3) ligand coverage, while enforcing charge neutrality. The models were relaxed with the DFT level of theory using VASP, generating a training set of 56,647 converged ionic steps. MatterSim-v1.0.0-1M was fine-tuned via the MatterTune package^29^ and validated against the test set using radial/angular distribution functions and Γ-point phonon density of states as validation metrics.

For this work, MatterSim-v1.0.0-1M^20^ pretrained machine learning potential was selected for fine-tuning because conservative models, in which forces are obtained as derivatives of the energy with respect to atomic positions, have demonstrated strong performance in phonon prediction.^30^ We first assessed the predictive performance of the vanilla MatterSim (hereafter referred to as the pretrained model) on nanocrystal configurations sampled from DFT geometry relaxation trajectories, using DFT energies and forces as references. For per atom energies, the pretrained model already achieves high accuracy with a near-unity *R* ^2^ and an RMSE of 0.0229 eV/atom (Figure 2a, left). However, its force predictions were highly unreliable with an *R* ^2^ of merely 0.3694. The culprit for such a low correlation was the low-force regime, as illustrated in the zoomed-in parity plot (Figure 2a, right). Since our data set comes from geometry relaxation, the vast majority of the force data points are clustered near equilibrium. In this region, the pretrained model completely loses its predictive power, with noticeable deviations centered around zero that render it unsuitable for phonon calculations. This behavior is expected because universal MLIPs are mainly trained on bulk crystals, where local environments can differ substantially from those at nanocrystal interfaces. Fine-tuning on our nanocrystal data set was therefore not only necessary but also highly effective. Both energy and force predictions improved by an order of magnitude relative to the baseline model. In particular, force accuracy in the near-equilibrium region increased dramatically, achieving an *R* ^2^ of 0.9878 and RMSE of 0.0230 eV/Å (Figure 2b, middle and right). This demonstrates that local environment learning via message-passing embedded in many machine learning potential models is transferable to nanocrystal systems, and that fine-tuning substantially enhances force prediction fidelity for further downstream tasks.

**Figure 2:**
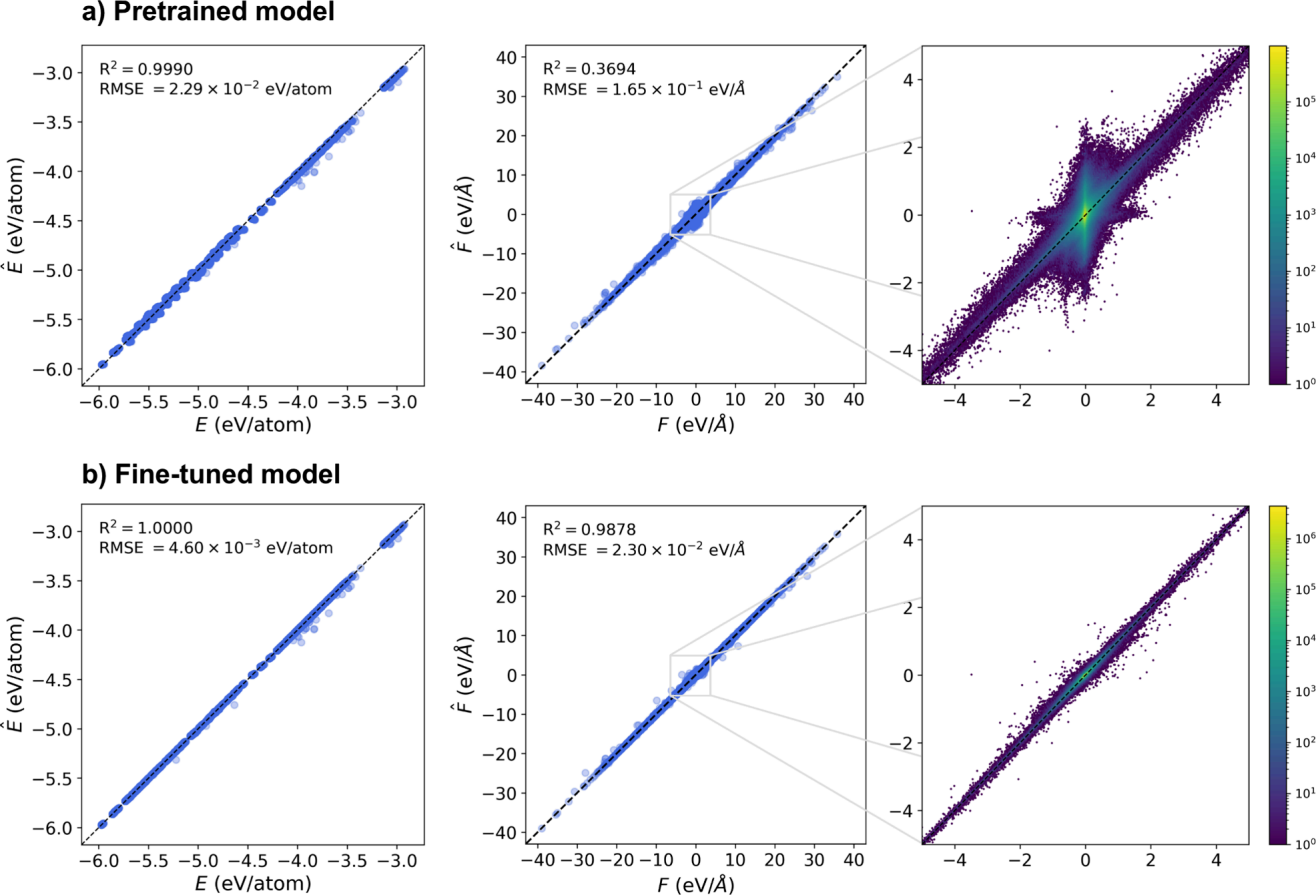
Test data set parity plots of predicted vs reference energies, *E*, and forces, *F*, for (a) the pretrained model and (b) the fine-tuned model. The left panels show energy predictions, the middle panels show force predictions, and the right panels present zoomed-in force parity plots with point density indicated by the color bar.

To further validate the fine-tuned MLIP (hereafter, MLIP), we compared its structural and vibrational predictions against DFT benchmarks (Figure 3). The Pb–Br radial distribution function (RDF) and Pb–Br–Pb angular distribution function (ADF), obtained from short NVT trajectories of 2 × 2 × 2 NC capped with 50% benzoate (BzO) and methylammonium (MA), show excellent agreement between the two methods (Figure 3a,b). Both methods have nearly identical positions and relative intensities of the major peaks characteristic to orthorhombic CsPbBr_3_,^31–33^ indicating that the MLIP can well reproduce the octahedral tilting in CsPbBr_3_ NCs at room temperature. The orthorhombic nature in the Pb–Br–Pb ADF was consistently observed across different supercell sizes including the extrapolative cases (Figure S1). We also assessed vibrational properties by computing the Γ-point phonon density of states (DOS) for a 3 × 3 × 3 bare NC as shown in Figure 3c. A linear force–displacement relationship in the harmonic regime was accurately captured by the MLIP, allowing us to compute phonon DOS via the finite difference method (Figure S2). The overall shape of the predicted DOS agrees nicely with the DFT reference, although noticeable peak shifts are present. Similar observations have been reported in other studies,^30,34^ underscoring the difficulty of accurately learning the Hessian, even when force predictions are reliable. In our case, the shifts likely arise from the discrepancies between the bulk and nanocrystal environments that influence the implicitly learned curvature of the potential energy surface. Despite the shifts, the MLIP well reproduces the relative intensities of characteristic low-energy modes, typically associated with PbBr_6_^4–^ octahedral rotation (M1) and Pb–Br–Pb stretching (M2 and M3) reported in other computational^10,13,35,36^ and experimental^11^ studies. Interestingly, we find that compared to the DFT peak positions, MLIP peak positions align better with the previously reported values (M2 ∼ 100 cm^–1^ and M3 ∼ 129 cm^–1^).^13,32^ These results confirm that the fine-tuned MLIP provides good accuracy in structural and vibrational predictions for both bare and ligand-passivated NCs for further analysis.

**Figure 3:**
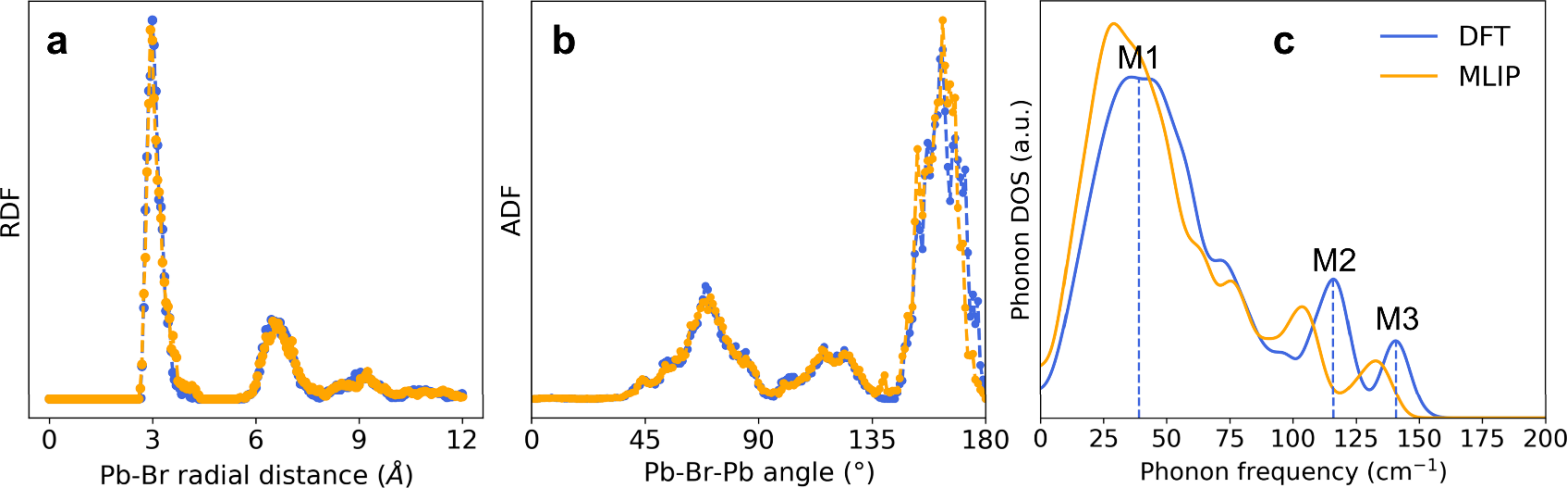
Validation of the fine-tuned MLIP against DFT. (a) Pb–Br radial distribution function, and (b) Pb–Br–Pb angular distribution function, obtained from 2 ps NVT (300 K) trajectories of 2 × 2 × 2 50% BzO/MA-capped NC. (c) Γ-point phonon DOS of 3 × 3 × 3 bare NC, computed via the finite difference method.

### Ligand-Controlled Phonon Dynamics

We systematically examined the ligand-dependent phonon modes in CsPbBr_3_ NCs to reveal how surface chemistry influences low-energy lattice dynamics (Figure 4). Phonon DOS was computed for NCs ranging from 3 × 3 × 3 (1.79 nm) to 7 × 7 × 7 (4.17 nm) with four representative surface passivation: (1) bare, (2) MA-capped, (3) BzO-capped, and (4) BzO/MA-capped. BzO was chosen as a model anionic ligand as it represents the broader family of carboxylic acid, one of the most widely used ligands in the colloidal synthesis of perovskite nanocrystals.^37^ In addition, BzO has demonstrated excellent passivation capability in our previous study,^16,38^ as well as in other reported works,^7,39^ making it a suitable model ligand for investigating ligand-induced effect on phonon modes. In all ligand-capped systems, ligand coverage was fixed at 50%, which is close to the upper bound^17,40–41^ of realistic ligand densities in colloidal halide perovskite NCs. The overall characteristics of the phonon DOS were largely size-independent for both bare^13^ and ligand-capped NCs. Across all systems, the Pb/Br partial DOS (dotted) shows that the M2 and M3 modes strictly arise from Pb–Br interactions. On the other hand, the M1 mode is accompanied by the contribution from the Cs atoms, indicating that the PbBr_6_^4–^ motion is strongly coupled with Cs^+^.^42^ Upon ligand passivation, the M2 mode went through broadening while M3 stayed relatively well-defined.

**Figure 4:**
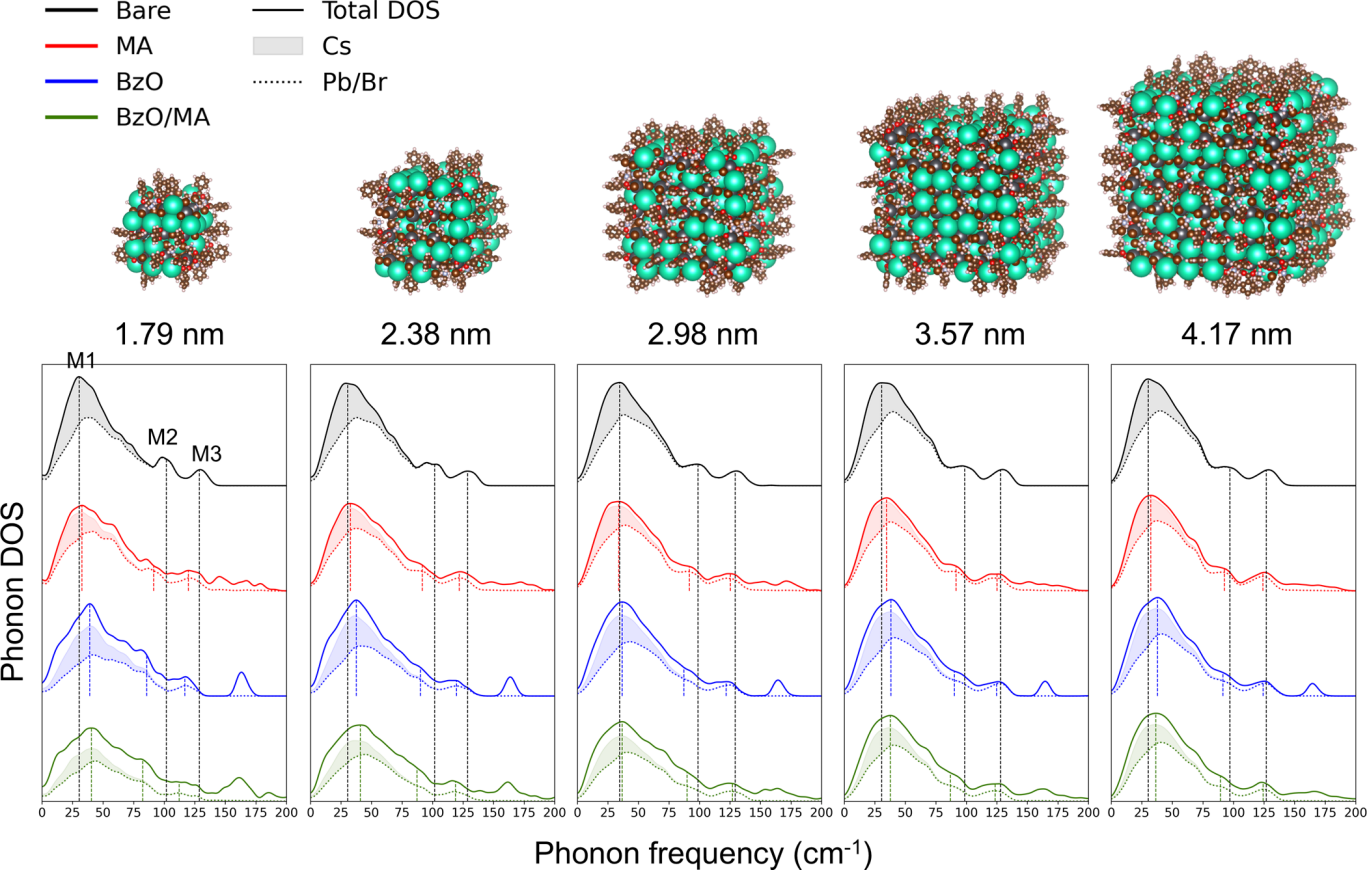
Ligand-dependent phonon DOS in CsPbBr_3_ NCs. Top: representative structures of MA and benzoate-capped NCs ranging from 3 × 3 × 3 to 7 × 7 × 7 supercells. Bottom: phonon DOS corresponding to each size for bare (black), methylammonium (MA, red), benzoate (BzO, blue), and BzO/MA (green) passivation. The solid lines represent the total DOS of the system, the dotted lines represent the partial DOS of Pb/Br, and the shaded regions represent the partial DOS of Cs. The vertical dashed lines indicate intensity-weighted peak positions of the low-energy modes (M1, M2, M3) for each system.

When compared to bare surfaces, introducing ligands causes significant redshifts of the Pb–Br–Pb stretching modes (M2 and M3), as evident from the downshift of Pb/Br partial DOS (dotted) in Figure 4. This behavior was consistently observed regardless of the types of surface ligands (i.e., anionic, cationic, or a mix of both), but the magnitude of redshift was generally larger for NCs containing anionic ligands (BzO or BzO/MA) compared to NCs only containing cationic ligands (MA), as well depicted in Figure 5a. The ligand-induced redshift of Pb–Br–Pb stretching is physically coherent with the nature of chemical bonding. The electron-withdrawing property of anionic ligands pulls away the electron density around the central Pb atom, weakening the neighboring Pb–Br interactions, which is analogous to reducing the spring constant in a harmonic oscillator. As a result, the vibrational frequency of the Pb–Br–Pb stretching reduces, which appears as a downshift of M2 and M3. While MA does not directly bond to Pb or Br, its NH_3_^+^ group can form hydrogen bonds with the surface halides,^9,43^ as observed in our relaxed structures (Figure S3a). This could have a similar effect in softening the Pb–Br bond although its effect would be smaller than that of the anionic ligands.

**Figure 5:**
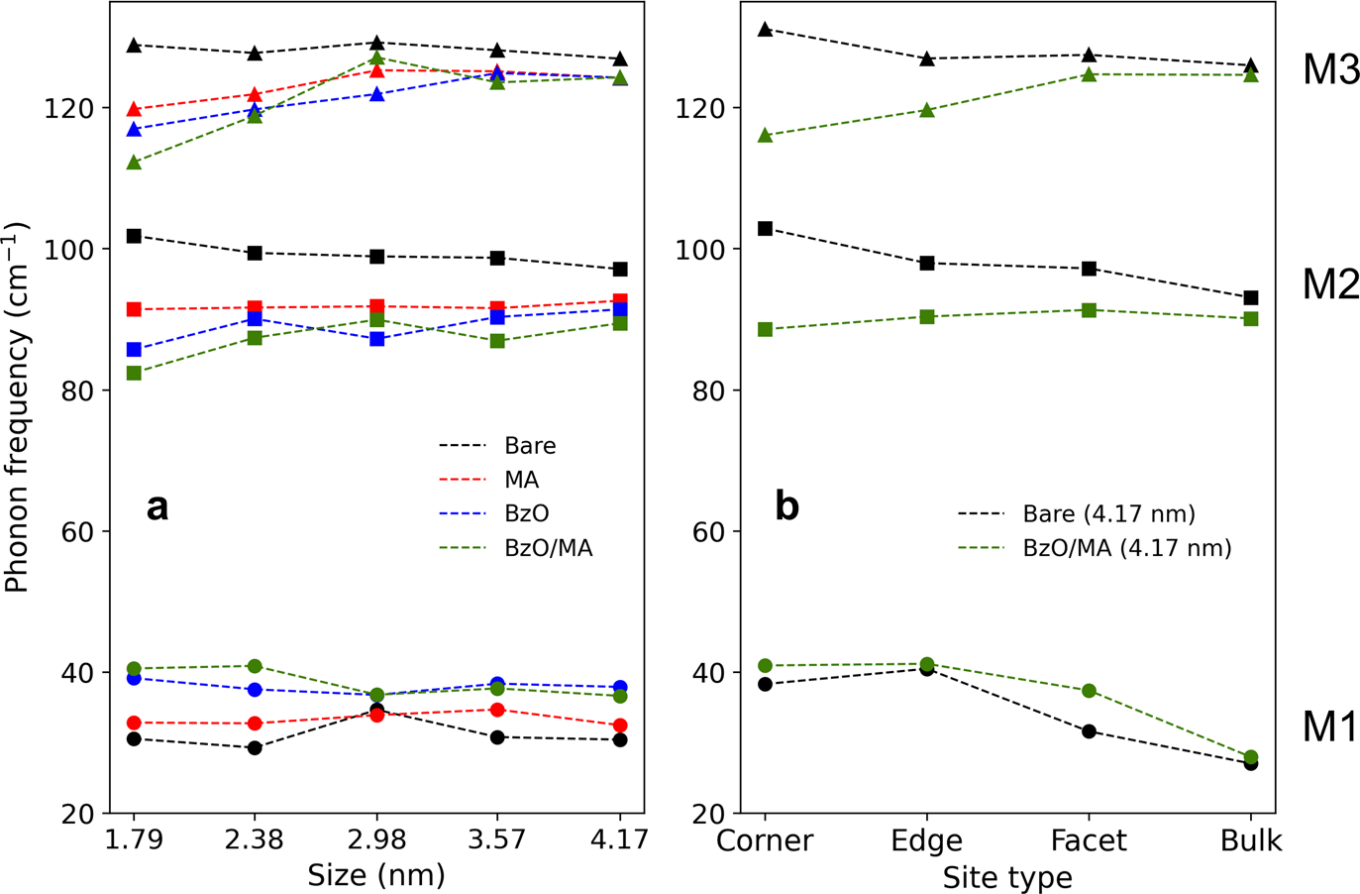
(a) The intensity-weighted peak positions of M1 (circle), M2 (square), and M3 (triangle) across systems with varying sizes and ligand passivation. Black, bare; red, MA; blue, BzO; green, BzO/MA-capped NCs. Ligands induce redshift of the Pb–Br–Pb stretching modes (M2, M3) and blueshift of the PbBr_6_^4–^ rotation mode (M1). The effect was generally larger for anionic ligands (blue, green) than cationic ligands (red). (b) The site-resolved partial DOS of the 4.17 nm bare (black) and BzO/MA-capped NCs (green). For M2 and M3, site type largely affects the redshift magnitude, with an increasing magnitude from bulk, facet, edge, to corners. In contrast, the blueshift of the lower-frequency M1 mode appears less site-dependent, likely because this mode contains mixed contributions from both Cs and Pb–Br motions, which attenuates the site-specific trend.

In the BzO/MA-capped systems, the hydrogens in the −NH_3_^+^ group can additionally interact with the BzO (Figure S3b). Additional to the good accuracy in the low-frequency regime, the MLIP could also reliably capture the downshift of the N–H stretching mode of MA at ∼3000 cm^–1^ in the presence of BzO (Figure S4), indicating a strong hydrogen bonding interaction between MA and the BzO on a copassivated surface. The bond softening effect of BzO and MA can take place simultaneously in BzO/MA-capped NCs. Hence, the mixed ligand NCs generally have the largest redshifts of M2 and M3. The MA–BzO hydrogen bond also perturbs the ligand-localized mode at ∼165 cm^–1^, which consistently appears across all sizes in the BzO-capped NCs. The partial phonon DOS of C, H, O, and N (Figure S5) confirms that this peak strictly pertains to BzO, likely involving out-of-plane bending motion.^44^ With the introduction of MA, a significant reduction and broadening of the C and O peaks occur, suggesting a spectral shift of the mode mediated by strong MA–BzO interactions. The role of low-energy ligand modes in optoelectronic properties remains unclear; however, we show that these modes can be effectively stabilized with synergistic surface cation–anion interactions.

The observed redshifts of the M2 and M3 modes indicate that Pb–Br vibrations are highly sensitive to the local environment, with ligands of different chemistry effectively modulating the force constants of the local bond stretching. Consistent with this picture, similar trends are observed across sites with different ligand coordination numbers, namely corners, edges, facets, and bulk sites. As shown in Figure 5b, the site-resolved partial DOS of the 4.17 nm NCs with bare and BzO/MA passivation reveals that the M2 and M3 redshifts are primarily driven by the surface sites, with the magnitude increasing from bulk to facet to edge to corner sites. This behavior is consistent with the site-dependent coordination of Pb atoms: Pb atoms at bulk, facet, edge, and corner sites can be coordinated by 0, 1, 2, and 3 surface ligands, respectively, resulting in a gradually stronger effect of Pb–Br bond softening with increasing ligand interactions. While slab models can provide useful insight into ligand–core interactions at the ideal (001) facet sites, a comparison between slab and nanocrystal models (Figures S6 and S7) shows that the ligand-induced phonon shifts are substantially smaller in slab models due to the absence of corners and edges. Therefore, full nanocrystal models are required to capture the distinct contributions of local surface coordination environments to the low-frequency phonon modes.

Interestingly, the octahedral rotation mode (M1) shows the opposite trend, exhibiting a clear blueshift upon ligand passivation. Consistent with the behavior of M2 and M3, BzO-capped NCs display larger M1 blueshifts than the MA-capped NCs. A similar ligand-induced stiffening of rotation modes was previously reported for FAPbBr_3_ and attributed to the multipodal hydrogen bonding and van der Waals interactions between surface FA and bulky ligands.^10^ Here, we find that even relatively small ligands such as BzO or MA are sufficient to induce a visible M1 blueshift. In nanocrystals, the pinning effect induced by ligand–ligand steric hindrance would be greater at the corner and edge-site Pb atoms, where multiple ligands can coordinate. Because M1 corresponds to a collective, low-frequency motion of many atoms, restricting the motion of just a few key surface sites can significantly suppress this global deformation. Likewise, in MA-capped NCs, a dense network of N–H···Br bridge bonds can similarly anchor surface atoms, stiffening the lattice and driving the observed blueshift. In contrast to the M2 and M3 modes, the blueshift of the M1 mode appears less site-dependent. This may arise from the mixed Cs and Pb–Br character of the mode, which can attenuate the apparent site-specific trend, rather than indicating that the sites themselves are insensitive.

To summarize, our results reveal a clear mode and site-selective response of CsPbBr_3_ nanocrystals to surface passivation. Local Pb–Br–Pb stretching modes redshift through ligand-induced softening of Pb–Br bonds and are strongly influenced by the local surface coordination environment, particularly at edge and corner sites. A more global octahedral rotation mode is stiffened through steric pinning and hydrogen-bond-mediated anchoring at surface lattice sites. The contrasting redshift–blueshift behavior highlights the dual role of ligands: they electronically weaken local metal-halide bonding while simultaneously imposing structural constraints that suppress low-energy lattice fluctuations. This interplay between local chemical bonding and global lattice rigidity illustrates how surface chemistry can be leveraged to selectively control lattice dynamics of halide perovskite nanocrystals.

### The Role of Ligand Binding Energies in M1 Blueshift

Dynamic lattice disorder in halide perovskites is closely linked to nonradiative losses through carrier quenching^45,46^ and strong electron–lattice interactions,^13,14,35^ which compromises the optical performance of halide perovskite NC-based applications. In particular, the low-energy octahedral rotation modes (M1) constitute a detrimental degree of freedom since photoexcitation couples strongly to Pb–X–Pb bond-angle distortions in the octahedra, which have been shown to accelerate phonon-assisted nonradiative recombination.^14^ In line with this, Kim et al. has shown that even a small shift in the lowest vibrational mode in the phonon DOS and experimental Raman spectra can substantially suppress lattice fluctuations and lead to a significant increase in PLQY.^10^ Taken together, these results suggest that ligand-induced blueshift of the M1 mode may be a key factor in suppressing nonradiative losses. Based on our observations that anionic ligands induce a larger shift, we further investigated the effect of different anionic ligands on realistic nanocrystal surfaces. In addition to benzoate (BzO), we considered thiophenolate (PhS) and phenylphosphonate (PhP), which share a benzene backbone but differ in their binding motifs and binding energies to surface Pb atoms (Figure S8). Figure 6 shows the intensity-weighted peak positions of the M1 mode in PhS/MA, BzO/MA, and PhP/MA-capped NCs. The introduction of anionic ligands induced blueshifts of the M1 mode compared to the bare surface baseline (black). Interestingly, we observed that the magnitude of the blueshift did not follow a monotonic trend with the ligand binding energy. In fact, BzO, which has an intermediate binding energy closest to the native Br atom, consistently induced the most blueshift across all sizes, compared to the stronger-binding PhP or weaker-binding PhS. The same trend was also observed in the NCs passivated only with anionic ligands (Figure S9), suggesting a systematic effect of the anionic ligands on the M1 blueshift. This result is consistent with our previous study, which revealed a nonmonotonic relationship between experimental PLQY and ligand binding energies, with BzO showing a higher PLQY than PhP and PhS.^38^ Given that the size and the steric pinning effect of the three anionic ligands are comparable, we conclude that the blueshift of the PbBr_6_^4–^ rotation mode is sensitive to the chemical properties of the anionic ligands coordinating the Pb atoms.

**Figure 6:**
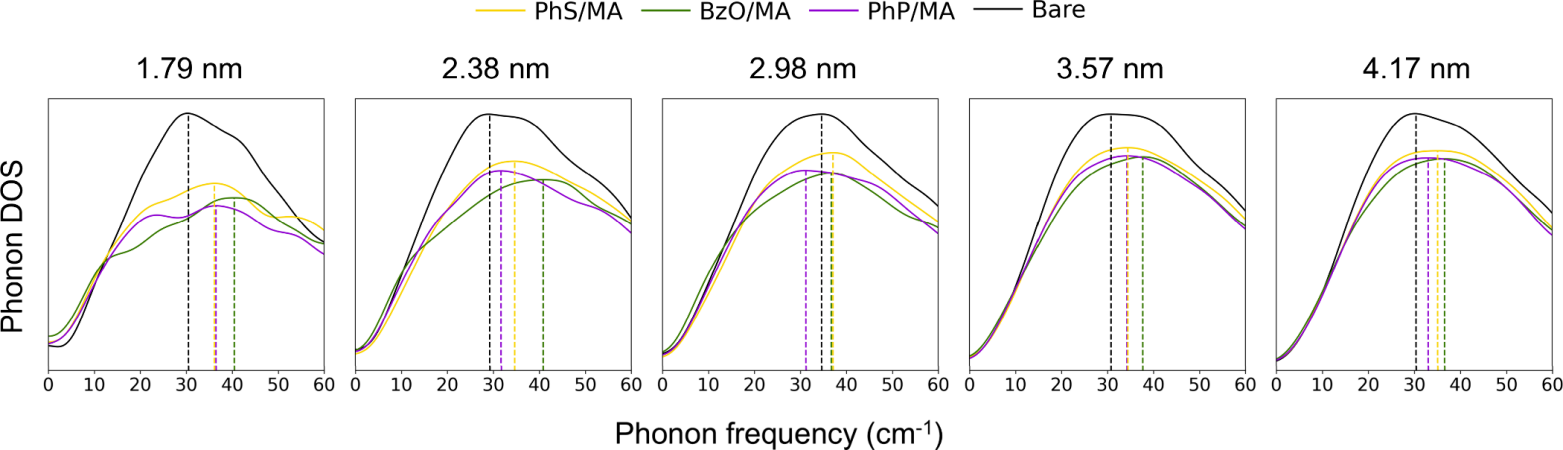
M1 mode in CsPbBr_3_ NCs with bare surface (black) and 50% coverage PhS/MA (yellow), BzO/MA (green), and PhP/MA (purple). The vertical dashed lines show the intensity-weighted peak position of the M1 mode. The ligand-capped NCs show consistent blueshift across all sizes compared to the bare NCs. BzO induces the largest blueshift systematically throughout all systems studied.

To gain further mechanistic insights into this nonmonotonic trend, we performed room-temperature molecular dynamics simulations for small 3 × 3 × 3 mixed-ligand NCs, where most atoms reside on the surface. We then computed the root mean squared fluctuations (RMSF) of the Pb atoms, which sit at the center of the PbBr_6_^4–^ octahedra (Figure 7). Notably, the BzO/MA system exhibited both the lowest average RMSF and the narrowest fluctuation distribution, indicating that the Pb atoms are more spatially constrained. In contrast, PhS/MA and PhP/MA-capped NCs showed larger average deviations and broader error bars, suggesting greater dynamical disorder within the inorganic framework. This observation aligns with our phonon calculations and can be rationalized by ligand-induced modulation of the local bonding environment discussed above: when the ligand–Pb interaction is either too weak or too strong relative to the intrinsic Pb–Br bonding, it could disrupt lattice equilibrium and enhances atomic motion. Weak binding leaves surface Pb sites insufficiently stabilized, whereas overly strong binding overconstrains or distorts neighboring Pb–Br bonds, both leading to excessive bond length fluctuations. Ligands with intermediate binding strength, comparable to that of the native halides, best preserve this balance, maintaining uniform Pb–Br interactions and minimizing lattice disorder. In summary, we show that even a modest blueshift in the global octahedral rotation mode can translate to significant suppression of dynamic fluctuations of soft, anharmonic halide perovskite lattices. Our work suggests the importance of ligand–core interactions and their nonmonotonic trend in modulating low-energy phonon modes, which can have broader implications for optoelectronic behaviors in these materials.

**Figure 7:**
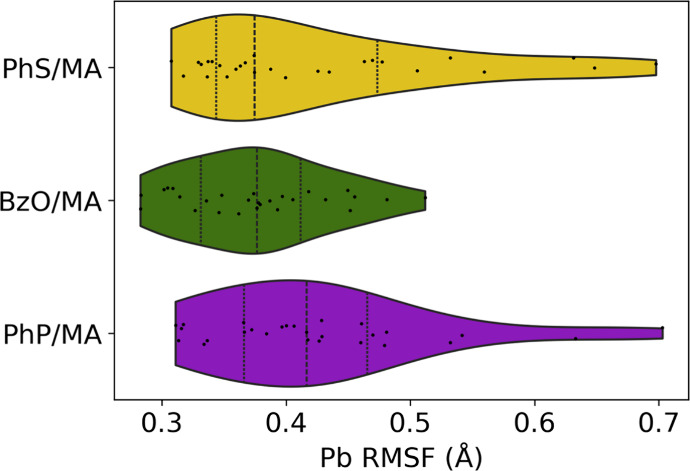
Root mean squared fluctuations (RMSF) of the surface Pb atoms in the representative 3 × 3 × 3 systems, obtained from the 40 ps NVT trajectories at 300 K.

## Conclusions

In conclusion, we have shown that surface ligands play a critical role in modulating low-energy phonons of CsPbBr_3_ nanocrystals. By fine-tuning a universal pretrained MLIP on small, ligand-passivated NCs, we systematically explored realistic NC sizes and diverse ligand chemistries, which were previously infeasible with conventional ab initio methods. We uncover a mode and site-dependent response, in which local Pb–Br–Pb stretching (M2, M3) softens due to ligand-induced weakening of Pb–Br bonds while the collective octahedral rotation (M1) stiffens via steric pinning and hydrogen-bond anchoring, with larger response coming from corner and edge sites present in finite nanocrystals. The cationic and anionic ligands can have synergistic effects on the surface by forming hydrogen bonds, which help stabilize ligand-localized low-energy modes and strengthen lattice stiffening. The M1 blueshift exhibits a nonmonotonic dependence on anionic ligand binding energy: BzO, which has the closest binding energy to native halides, most effectively damps rotational disorder, whereas much stronger (PhP) or weaker (PhS) binding ligands could be suboptimal, as corroborated by molecular dynamics. Our work provides insights into ligand-controlled phonon dynamics in realistic halide perovskite nanocrystals, which have important implications for the radiative performance of these materials. More broadly, our work establishes fine-tuned MLIP as a powerful tool to bridge theoretical insights and practical design rules for next-generation perovskite nanocrystal optoelectronics.

## Supplementary Material



## Data Availability

The fine-tuned MatterSim model, the training data set, representative atomistic models of ligand-passivated CsPbBr_3_, and sample analysis codes are available at https://github.com/Hu-group-at-GT/cspbbr3-nc-phonon-mlip.

## Notes

Computational details; ligand structures and binding energies; structural analyses; additional phonon density of states; and site-dependent results (PDF)
