# Supplementary material for: Ligand-Controlled Phonon Dynamics in CsPbBr3 Nanocrystals Revealed by Machine-Learned Interatomic Potentials
Source: JACS Au. 2026 Apr 27;6(5):3039–47. doi: 10.1021/jacsau.6c00434 (PMC13213489; doi:10.1021/jacsau.6c00434)
Supplement: Supplementary file 1 [file au6c00434_si_001.pdf]

Online Supporting Information for

Ligand-Controlled Phonon Dynamics in $CsPbBr_3$ Nanocrystals Revealed by Machine-Learned Interatomic Potentials

Seungjun Cha,[1] Chen Wang,[2,3,*] Victor Fung,[4,*] Guoxiang Hu[1,5,*]

[1] School of Materials Science and Engineering, Georgia Institute of Technology, Atlanta, GA 30332, USA

[2] Department of Chemistry and Biochemistry, Queens College, City University of New York, New York, NY 11367, USA

[3] The Graduate Center, City University of New York, New York, NY 10016, USA

[4] School of Computational Science and Engineering, Georgia Institute of Technology, Atlanta, GA 30332, USA

[5] School of Chemistry and Biochemistry, Georgia Institute of Technology, Atlanta, GA 30332, USA

*Corresponding author.

Email: chen.wang@qc.cuny.edu, victorfung@gatech.edu, emma.hu@mse.gatech.edu

METHODS

1. Model generation

Cubic $CsPbBr_3$ nanocrystals (NCs) with dimensions of $n \times n \times n$ unit cells (n = 3, 4, 5) were constructed with a lattice parameter of 5.95 Å using Pymatgen[1] (v2024.11.13). All surfaces were initially terminated with Cs and Br atoms[2,3]. The net charge of each NC was obtained by summing over the formal ionic charges of Cs^+, Pb^{2+}, and Br^-. For charge neutrality, Cs atoms were first removed from all corner sites, which had the lowest binding energies (Table S2), and then randomly deleted from remaining sites as needed. Organic ligands were generated from their SMILES representations and optimized with the Merck Molecular Force Field (MMFF)[4] as implemented in RDKit[5] (v2024.03.5). Each ligand was oriented such that its binding motif was perpendicular to {100} for face sites, {110} for edge sites, and {111} for corner sites, if any, while retaining rotational freedom about the surface normal. Cationic ligands were used to replace surface Cs sites, and anionic ligands replaced surface Br sites, to achieve specified coverages ranging from 20% to 60%. For mixed-ligand NCs, cationic–anionic ligand pairs were placed adjacently[6]. After ligand placement, a large vacuum of 15 Å was introduced along all three Cartesian directions to avoid interactions between periodic images. A random perturbation proportional to the covalent radius of each atom was applied: 10–15% for C, H, O, N, S, and P, and 15–25% for Cs, Pb, and Br. For mixed-ligand NCs, a short pre-relaxation was performed using the pretrained MatterSim-v1.0.0-1M[7] without fine-tuning until the maximum force was below 0.5 eV/Å to prevent steric crowding prior to density functional theory (DFT) geometry relaxation. In total, 236 NC models were generated with varied supercell sizes, ligand coverages, and surface passivation schemes.

2. DFT geometry relaxation

Density Functional Theory (DFT) calculations were performed using the Vienna *Ab initio* Simulations Package[8] (VASP, v.6.4.2). The ion-electron interaction was described by the projector-augmented wave (PAW) method[9], with a plane-wave cutoff energy set to 400 eV. The exchange-correlation interaction was described by the generalized gradient approximation (GGA) method of Perdew, Burke, and Ernzerhof (PBE)[10]. All nanocrystal (NC) models were treated non-periodic with a large vacuum of 15 Å in all directions, hence a Γ-point (1 × 1 × 1) Monkhorst-Pack *k*-point mesh was used. Spin–orbit coupling (SOC) was ignored in these calculations. The D3 dispersion energy-corrections were not included at this stage but in the later steps *post hoc* (See Section 4). The energy convergence criterion of 1×10^{-4} eV and the force convergence criterion of 0.03 eV/Å were used. The precision and electronic minimization algorithms were set to PREC = Normal and ALGO = Fast, respectively. Due to the large computational cost of relaxing NC models, each structure was relaxed either until full convergence or until a set wall-time limit was reached, whichever occurs first. As a result, some NCs were only partially relaxed during the dataset generation. All validation cases were converged fully. On average, each run went through ~240 ionic steps. All calculations were performed using 8 NVIDIA A100 GPUs per run.

3. Fine-tuning machine learning potential

56,647 structural snapshots that electronically converged were extracted from the DFT geometry relaxation trajectories. The dataset was randomly split into training (68%), validation (12%), and test (20%) sets. Only total energies and atomic forces were included. All layers of the MatterSim-v1.0.0-1M checkpoint were fine-tuned using the MatterTune[11] (v.0.1.0) package. The two-body cutoff and three-body cutoffs were set as 6.5 Å and 4.0 Å, respectively, to sufficiently capture the

Pb–Br bonds and Pb–Br–Pb angles within the radial cutoffs. The remaining graph conversion hyperparameters were set to the MatterSim default. The loss function was the mean squared error (MSE) of energies and forces with the relative weight of 1 and 10, respectively. The model was optimized using AdamW[12] with the initial learning rate 1×10^{-4} and the cosine annealing as a scheduler (final learning rate of 1×10^{-6}). Training was done for 350 epochs with batch size 8 on 4 NVIDIA V100 GPUs. The best model was selected based on the lowest validation mean absolute error (MAE) of atomic forces.

4. Molecular dynamics

Ab initio molecular dynamics simulations were performed in the canonical ensemble (NVT) using the Langevin thermostat as implemented in VASP. The target temperature was set to 300 K, and initial velocities were sampled from a Maxwell–Boltzmann distribution. A timestep of 1 fs was chosen in accordance with the highest vibrational modes of hydrogen. The Langevin damping constant was set to 20 ps^{-1} for all elements (LANGEVIN_GAMMA = 20) for quick thermal equilibration. The energy convergence criterion was tightened to 1×10^{-6} eV, and precision was set to PREC = Accurate for numerical stability. All other settings were consistent with the geometry relaxations. Due to the high computational cost of nanocrystal models, the duration was typically set to ~2 ps (2000 steps), which was sufficient to sample near-equilibrium phase space.

Molecular dynamics with fine-tuned MLIP was performed using the Atomic Simulation Environment[13] (ASE, v3.24.0) interface provided in MatterTune. For validation studies, all simulation settings were the same as those used for *ab initio* simulations. For long MD (~40 ps), D3 dispersion-energy corrections[14] with Becke and Johnson damping[15] were additively applied to stabilize energy drifts, using the Simple-DFTD3 package[16] and ASE SumCalculator. Snapshots

were saved every 50 fs and those in the first 10 ps were discarded for thermal equilibration. Only the remaining snapshots were used to compute radial distribution function (RDF) and angular distribution function (ADF) using Auto-FOX[17].

5. Phonon calculations

For the DFT workflow, we started from the DFT-relaxed geometries without further tightening the force convergence criteria, as stricter relaxation yielded only marginal differences in the phonon DOS while significantly increasing the computational cost (see Figure S10). Here, Phonopy[18] (v.2.38.0) was used to create displaced structures and the force constant matrix. Each atom in the relaxed structure was displaced by ± 0.01 Å in the *x, y, z* directions, creating $6N$ displaced structures, where N is the total number of atoms. For each displaced structure, a single-point DFT calculation was performed in VASP using the same settings as the geometry relaxations but with the tighter energy convergence criterion of 1×10^{-8} eV, PREC = Accurate, and ALGO = Normal for improved force precision. The force constant matrix was computed by the finite difference method as implemented in Phonopy.

We have made some changes to the ASE library to (1) accommodate VASP-calculated force constant in ASE for consistency with the MLIP-based workflow, and (2) allow proper calculation of phonon partial density of states weighted by atomic mass. This patch is publicly available at https://github.com/Hu-group-at-GT/cspbbr3-nc-phonon-mlip. Once the force constant matrix was obtained from Phonopy, it was passed to the modified ASE Phonons module to compute the Γ-point phonon DOS. The partial DOS was computed with the same module by projecting eigenvector amplitudes of the specified atoms onto the total DOS then normalizing by atomic mass. The phonon DOS was smoothed by assigning each eigenfrequency a normalized

Gaussian ($\sigma = 3.0\ cm^{-1}$) and summing over all modes. M2 and M3 peak positions were obtained from the intensity-weighted center using the following equation:

$$\omega_{\text{peak}} = \frac{\int_a^b \omega\, DOS(\omega)\, d\omega}{\int_a^b DOS(\omega)\, d\omega}$$

where ω is the phonon frequency, $DOS(\omega)$ is the phonon density of states, and a and b define the frequency window associated with each M2 and M3 mode. For M1 mode, a fixed frequency window of $\pm 10\ cm^{-1}$ centered on the peak maximum was used.

The ML-based phonon workflow was carried out entirely within ASE. In this case, the starting geometry was either taken directly after DFT geometry relaxation (for validation cases) or relaxed using the L-BFGS algorithm[20] with the fine-tuned MLIP. The subsequent displacement, force constant computation, and DOS calculation followed the same procedures using the modified ASE Phonons module. The D3 dispersion was ignored during the phonon workflow because the force contribution of the D3 dispersion at finite displacements around the equilibrium point were orders of magnitude smaller than the MLIP forces.

Supplementary Figures and Tables

Table S1. A full list of anionic ligands used for modelling mixed-ligand NCs and their DFT-calculated binding energy to the 2×2×3 methylammonium-capped $CsPbBr_3$ slab.

Ligands	Molecular structure	Binding Energy (eV)
**Bromide (Br)**	Br	-2.01
**Octylphosphonate (OP)**	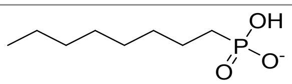	-2.65 (Strong)
**Phenylphosphonate (PhP)**	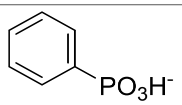	-2.67 (Strong)
**Dodecyl sulfate (DS)**	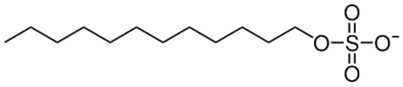	-2.60 (Strong)
**Dodecyl benzenesulfonate (DBS)**	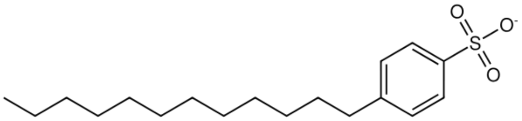	-2.63 (Strong)
**Benzenesulfonate (BS)**	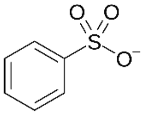	-2.33 (Strong)
**Pyrrole-2-carboxylate (Pyr-2-COO)**	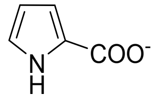	-1.86 (Medium)
**2-Furoate (2-Fur)**	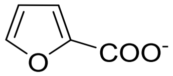	-1.92 (Medium)
**2-Thiophenecarboxylate (TPC)**	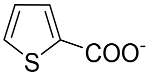	-1.92 (Medium)
**4-Bromothiophene-2-carboxylate (4-Br-2-TPC)**	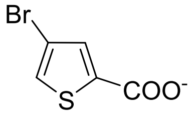	-2.01 (Medium)
**Benzoate (BzO)**	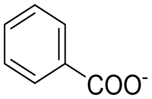	-2.20 (Medium)
**Isonicotinate (INC)**	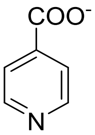	-2.16 (Medium)

**Anthranilate (ANT)**	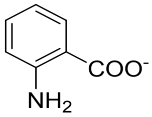	-1.81 (Medium)
**Pyridine (Py)**	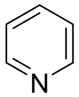	-0.79 (Weak)
**Thiophenolate (PhS)**	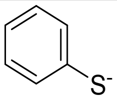	-0.87 (Weak)
**Thiophene (Th)**	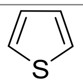	-0.47 (Weak)
**Anthranilic acid (2-ABA)**	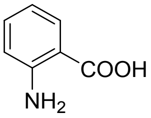	-1.08 (Weak)
**2,5-pyrazinedicarboxylic acid (PDC)**	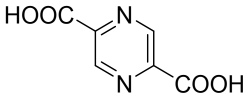	-0.86 (Weak)
**L-cysteine (Cys)**	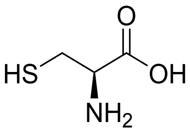	-1.03 (Weak)
**Glutathione (GSH)**	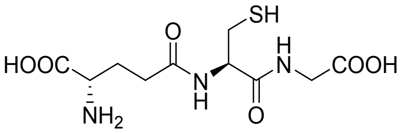	-1.09 (Weak)

Table S2. A site-dependent DFT-calculated binding energy of a Cs atom in a pristine, Cs-Br terminated $CsPbBr_3$ nanocrystal (supercell of 3×3×3)

	Corner	Edge	Face
Cs binding energy (eV)	-0.71	-1.05	-1.11

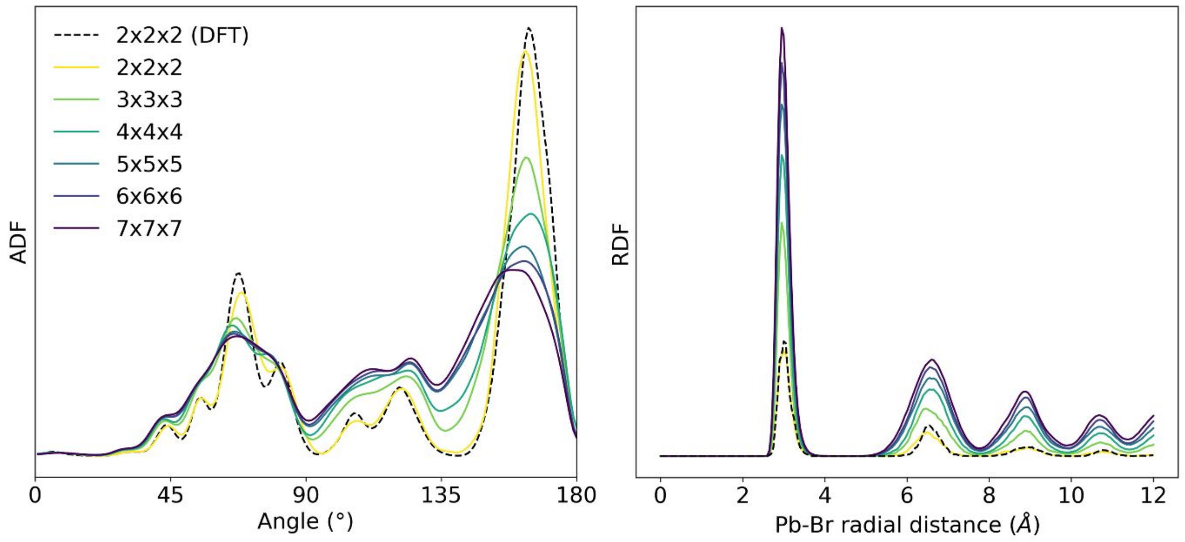

Figure S1. Angular distribution functions of bare $CsPbBr_3$ with varying supercell sizes (2×2×2 to 7×7×7), obtained from 10 ps NVT trajectories at 300 K. The fine-tuned MLIP well reproduced the octahedral tilting behavior of $CsPbBr_3$ at room temperature across all sizes.

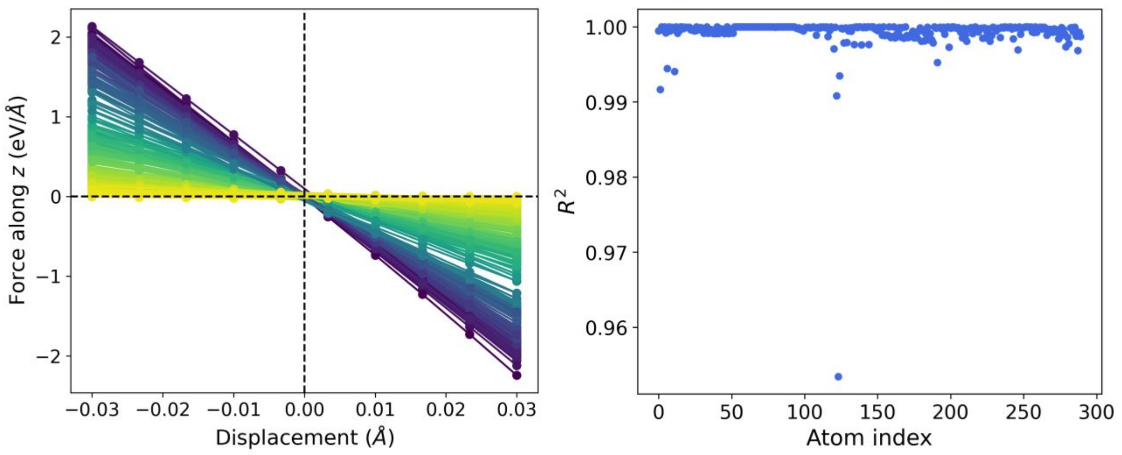

Figure S2. Harmonic force–displacement response computed from the fine-tuned MLIP for a 2×2×2 $CsPbBr_3$ capped with 50% BzO/MA. Each colored line (left) corresponds to the force response of a single atom under small displacements along the *z*-axis. The scatter plot (right) shows the R^2 of the linear fit for each line. The colormap is proportional to the slope of the fitted line and is shown only for visualization. The linear relation demonstrates that the fine-tuned MLIP can accurately capture the harmonic vibrational behavior, which is required for phonon DOS calculations via the finite difference method.

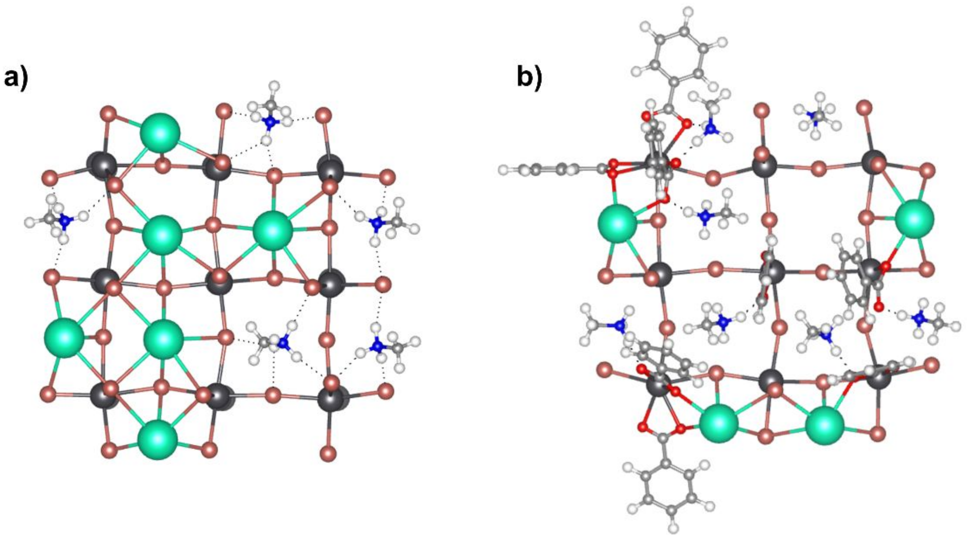

Figure S3. Optimized atomic structures of (a) MA-capped, and (b) BzO/MA-capped NC surfaces. H, white; C, light grey; N, blue; O, red; Br, brown; Pb, dark grey; Cs, green. Dotted lines represent hydrogen bonds between H–Br (a), or H–O (b).

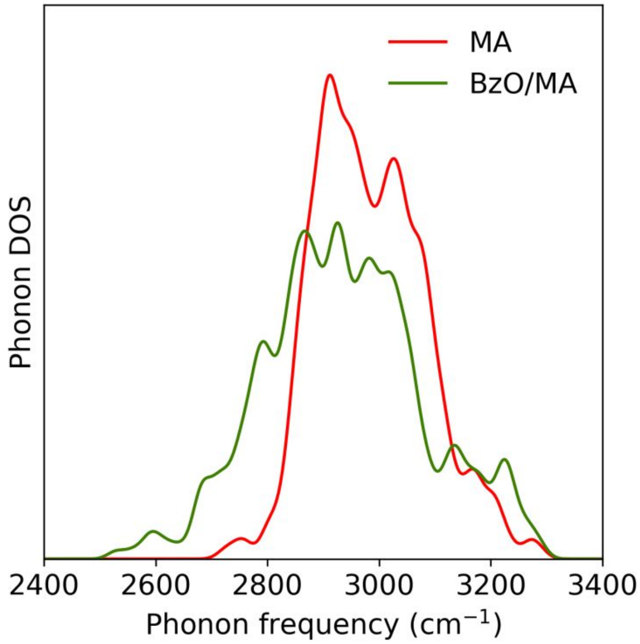

Figure S4. The N–H stretching mode in the 7×7×7 MA and BzO/MA-capped NCs. A clear downshift and broadening of the N–H stretching in the mixed-ligand NCs suggest a strong hydrogen bond between the cationic and anionic species.

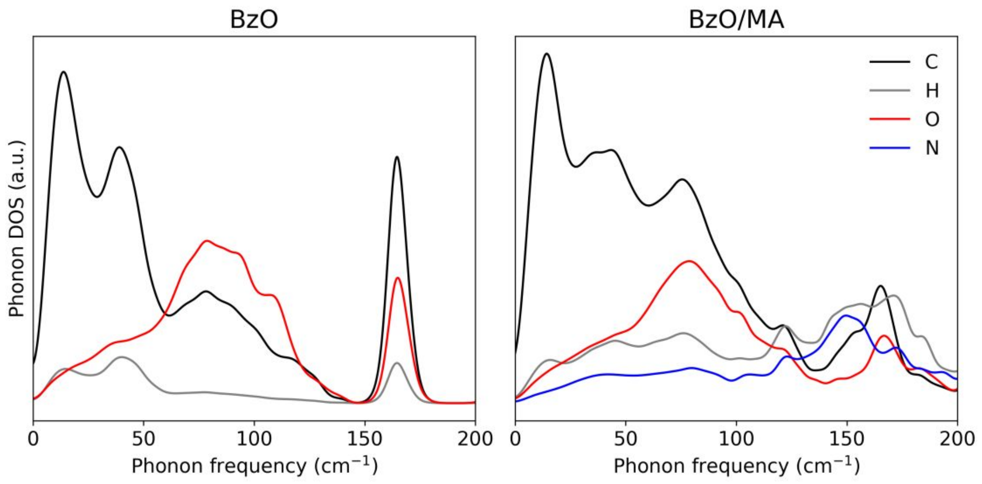

Figure S5. Partial DOS of C, H, O, and N for 7×7×7 MA and BzO/MA-capped NCs. A significant reduction and broadening of the C and O peaks are observed in the mixed-ligand NCs, suggesting a spectral shift of the mode mediated by strong MA–BzO interactions.

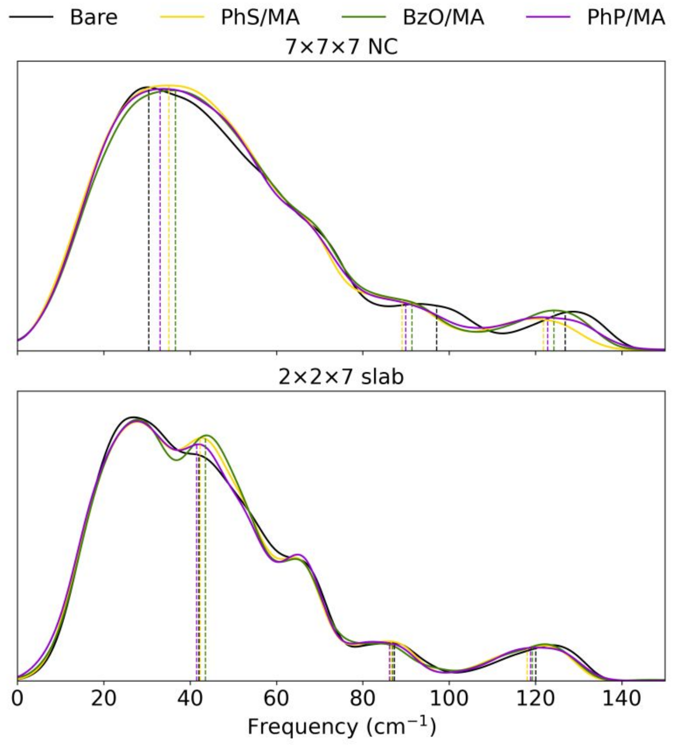

Figure S6. Comparison of the low-frequency phonon DOS of 7×7×7 nanocrystals (top) and 2×2×7 slab models (bottom) for bare, PhS/MA-, BzO/MA-, and PhP/MA-passivated $CsPbBr_3$. Dashed vertical lines indicate the intensity-weighted peak positions of the M1, M2, and M3 modes.

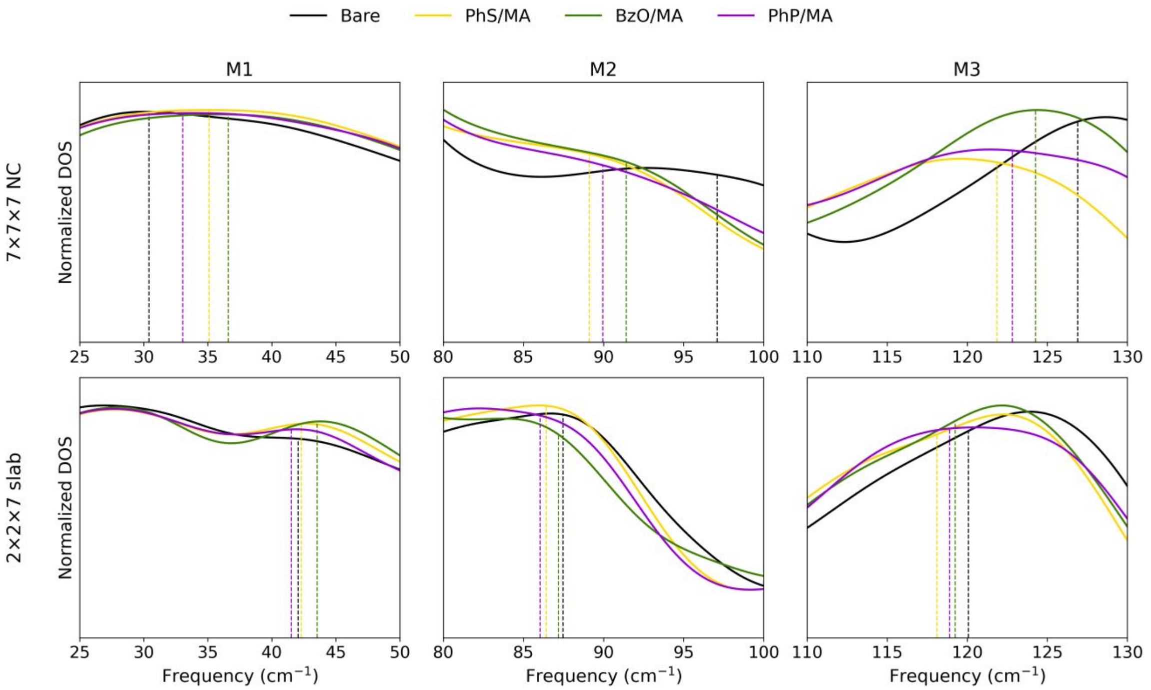

Figure S7. Zoomed-in views of the low-frequency phonon DOS in the M1, M2, and M3 spectral regions for 7×7×7 nanocrystals (top) and 2×2×7 slab models (bottom) of bare, PhS/MA-, BzO/MA-, and PhP/MA-passivated $CsPbBr_3$. Dashed vertical lines indicate the intensity-weighted peak positions of the M1, M2, and M3 modes. Although the slab models capture some qualitative ligand-dependent trends as the nanocrystal models, the corresponding peak shifts are markedly smaller.

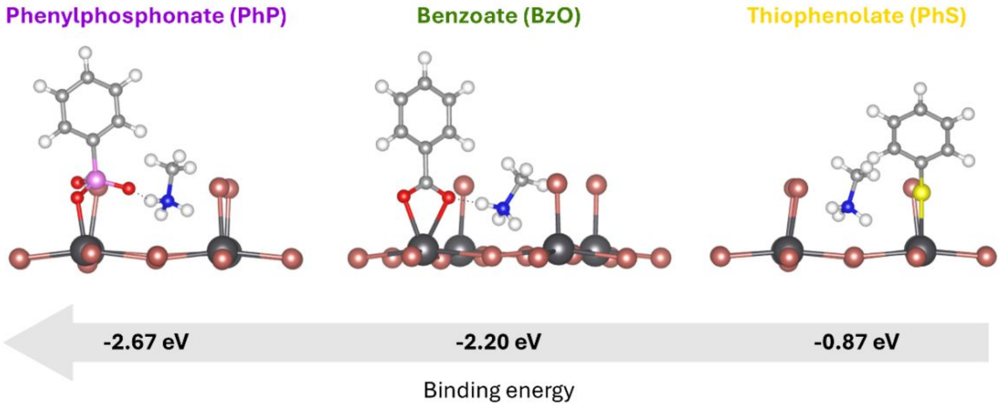

Figure S8. Optimized atomic structures of a $CsPbBr_3$ surface capped with PhP/MA (left), BzO/MA (middle), and PhS/MA (right). H, white; C, light grey; N, blue; O, red; P, pink; S, yellow; Br, brown; Pb, dark grey. The dotted line

represents hydrogen bonds. The DFT-calculated binding energy corresponds to that of each anionic ligand with respect to the 2×2×3 MA-capped $CsPbBr_3$ slab (also shown in Table S1).

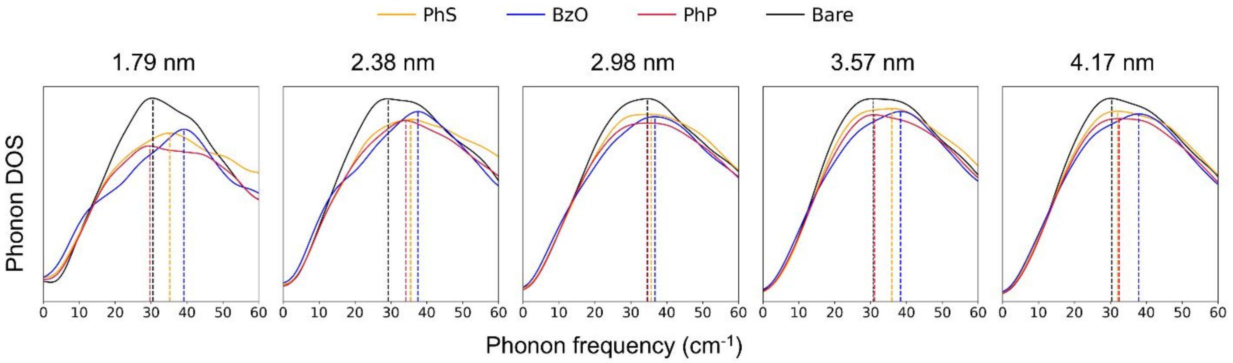

Figure S9. Ligand-dependent M1 mode in $CsPbBr_3$ NCs with 50% coverage PhS (orange), BzO (blue), PhP (brown). The vertical dashed lines show the intensity-weighted peak position of the M1 mode.

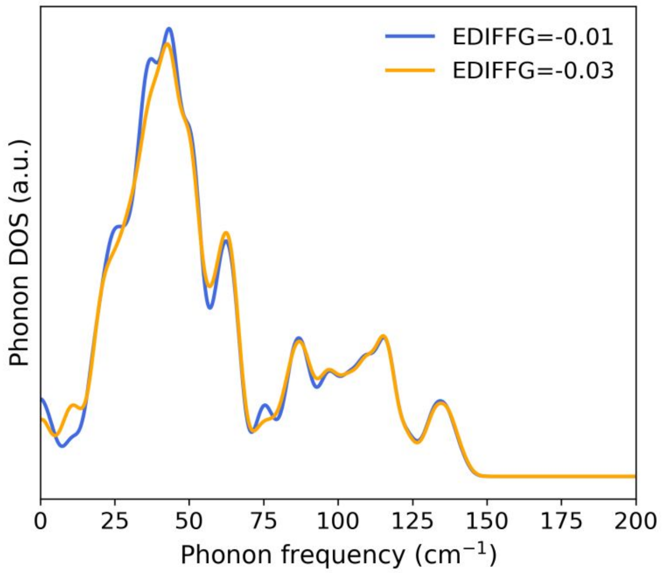

Figure S10. The *ab initio* phonon DOS of 2×2×2 bare $CsPbBr_3$ NC with the force criterion (EDIFFG) of -0.01 and -0.03. A stricter convergence criterion beyond -0.03 made negligible differences while adding significant computational cost for relaxing free-floating nanocrystals.

REFERENCES

(1) Ong, S. P.; Richards, W. D.; Jain, A.; Hautier, G.; Kocher, M.; Cholia, S.; Gunter, D.; Chevrier, V. L.; Persson, K. A.; Ceder, G. Python Materials Genomics (Pymatgen): A Robust, Open-Source Python Library for Materials Analysis. *Comput. Mater. Sci.* **2013**, *68*, 314–319. https://doi.org/10.1016/j.commatsci.2012.10.028.

(2) ten Brinck, S.; Zaccaria, F.; Infante, I. Defects in Lead Halide Perovskite Nanocrystals: Analogies and (Many) Differences with the Bulk. *ACS Energy Lett.* **2019**, *4* (11), 2739–2747. https://doi.org/10.1021/acsenergylett.9b01945.

(3) Chen, Y.; Smock, S. R.; Flintgruber, A. H.; Perras, F. A.; Brutchey, R. L.; Rossini, A. J. Surface Termination of CsPbBr3 Perovskite Quantum Dots Determined by Solid-State NMR Spectroscopy. *J. Am. Chem. Soc.* **2020**, *142* (13), 6117–6127. https://doi.org/10.1021/jacs.9b13396.

(4) Halgren, T. A. Merck Molecular Force Field. I. Basis, Form, Scope, Parameterization, and Performance of MMFF94. *J. Comput. Chem.* **1996**, *17* (5–6), 490–519. https://doi.org/10.1002/(SICI)1096-987X(199604)17:5/6<490::AID-JCC1>3.0.CO;2-P.

(5) RDKit: Open-Source Cheminformatics. https://www.rdkit.org/.

(6) Malinoski, A.; Hu, G.; Wang, C. Strong Bidentate Coordination for Surface Passivation and Ligand-Shell Engineering of Lead Halide Perovskite Nanocrystals in the Strongly Quantum-Confined Regime. *J. Phys. Chem. C* **2021**, *125* (44), 24521–24530. https://doi.org/10.1021/acs.jpcc.1c07952.

(7) Yang, H.; Hu, C.; Zhou, Y.; Liu, X.; Shi, Y.; Li, J.; Li, G.; Chen, Z.; Chen, S.; Zeni, C.; Horton, M.; Pinsler, R.; Fowler, A.; Zügner, D.; Xie, T.; Smith, J.; Sun, L.; Wang, Q.; Kong, L.; Liu, C.; Hao, H.; Lu, Z. MatterSim: A Deep Learning Atomistic Model Across Elements, Temperatures and Pressures. arXiv May 10, 2024. https://doi.org/10.48550/arXiv.2405.04967.

(8) Kresse, G.; Furthmüller, J. Efficient Iterative Schemes for Ab Initio Total-Energy Calculations Using a Plane-Wave Basis Set. *Phys. Rev. B* **1996**, *54* (16), 11169–11186. https://doi.org/10.1103/PhysRevB.54.11169.

(9) Kresse, G.; Joubert, D. From Ultrasoft Pseudopotentials to the Projector Augmented-Wave Method. *Phys. Rev. B* **1999**, *59* (3), 1758–1775. https://doi.org/10.1103/PhysRevB.59.1758.

(10) Perdew, J. P.; Burke, K.; Ernzerhof, M. Generalized Gradient Approximation Made Simple. *Phys. Rev. Lett.* **1996**, *77* (18), 3865–3868. https://doi.org/10.1103/PhysRevLett.77.3865.

(11) Kong, L.; Shoghi, N.; Hu, G.; Li, P.; Fung, V. MatterTune: An Integrated, User-Friendly Platform for Fine-Tuning Atomistic Foundation Models to Accelerate Materials Simulation and Discovery. *Digit. Discov.* **2025**, *4* (8), 2253–2262. https://doi.org/10.1039/D5DD00154D.

(12) Loshchilov, I.; Hutter, F. Decoupled Weight Decay Regularization. arXiv January 4, 2019. https://doi.org/10.48550/arXiv.1711.05101.

(13) Hjorth Larsen, A.; Jørgen Mortensen, J.; Blomqvist, J.; Castelli, I. E.; Christensen, R.; Dułak, M.; Friis, J.; Groves, M. N.; Hammer, B.; Hargus, C.; Hermes, E. D.; Jennings, P. C.; Bjerre Jensen, P.; Kermode, J.; Kitchin, J. R.; Leonhard Kolsbjerg, E.; Kubal, J.; Kaasbjerg, K.; Lysgaard, S.; Bergmann Maronsson, J.; Maxson, T.; Olsen, T.; Pastewka, L.; Peterson, A.; Rostgaard, C.; Schiøtz, J.; Schütt, O.; Strange, M.; Thygesen, K. S.; Vegge, T.; Vilhelmsen, L.; Walter, M.; Zeng, Z.; Jacobsen, K. W. The Atomic Simulation

Environment—a Python Library for Working with Atoms. *J. Phys. Condens. Matter* **2017**, *29* (27), 273002. https://doi.org/10.1088/1361-648X/aa680e.
(14)Grimme, S.; Antony, J.; Ehrlich, S.; Krieg, H. A Consistent and Accurate Ab Initio Parametrization of Density Functional Dispersion Correction (DFT-D) for the 94 Elements H-Pu. *J. Chem. Phys.* **2010**, *132* (15), 154104. https://doi.org/10.1063/1.3382344.
(15)Grimme, S.; Ehrlich, S.; Goerigk, L. Effect of the Damping Function in Dispersion Corrected Density Functional Theory. *J. Comput. Chem.* **2011**, *32* (7), 1456–1465. https://doi.org/10.1002/jcc.21759.
(16)Ehlert, S. Simple DFT-D3: Library First Implementation of the D3 Dispersion Correction. *J. Open Source Softw.* **2024**, *9* (103), 7169. https://doi.org/10.21105/joss.07169.
(17)van Beek, B. Auto-FOX, 2023. https://doi.org/10.5281/zenodo.7919377.
(18)Togo, A.; Chaput, L.; Tadano, T.; Tanaka, I. Implementation Strategies in Phonopy and Phono3py. *J. Phys. Condens. Matter* **2023**, *35* (35), 353001. https://doi.org/10.1088/1361-648X/acd831.
(19)Virtanen, P.; Gommers, R.; Oliphant, T. E.; Haberland, M.; Reddy, T.; Cournapeau, D.; Burovski, E.; Peterson, P.; Weckesser, W.; Bright, J.; van der Walt, S. J.; Brett, M.; Wilson, J.; Millman, K. J.; Mayorov, N.; Nelson, A. R. J.; Jones, E.; Kern, R.; Larson, E.; Carey, C. J.; Polat, İ.; Feng, Y.; Moore, E. W.; VanderPlas, J.; Laxalde, D.; Perktold, J.; Cimrman, R.; Henriksen, I.; Quintero, E. A.; Harris, C. R.; Archibald, A. M.; Ribeiro, A. H.; Pedregosa, F.; van Mulbregt, P. SciPy 1.0: Fundamental Algorithms for Scientific Computing in Python. *Nat. Methods* **2020**, *17* (3), 261–272. https://doi.org/10.1038/s41592-019-0686-2.
(20)Liu, D. C.; Nocedal, J. On the Limited Memory BFGS Method for Large Scale Optimization. *Math. Program.* **1989**, *45* (1), 503–528. https://doi.org/10.1007/BF01589116.
